# Integrating bulk and single-cell RNA sequencing data reveals epithelial-mesenchymal transition molecular subtype and signature to predict prognosis, immunotherapy efficacy, and drug candidates in low-grade gliomas

**DOI:** 10.3389/fphar.2023.1276466

**Published:** 2023-11-20

**Authors:** Chengcheng Wang, Zheng He

**Affiliations:** ^1^ Department of Pharmacy, Qilu Hospital (Qingdao), Cheeloo College of Medicine, Shandong University, Qingdao, Shandong, China; ^2^ Department of Neurosurgery, Qilu Hospital (Qingdao), Cheeloo College of Medicine, Shandong University, Qingdao, Shandong, China

**Keywords:** low-grade gliomas, Epithelial-mesenchymal transition (EMT), molecular subtypes, tumor microenvironment, immunotherpapy, signature, single cell RNA analysis

## Abstract

**Objective:** Epithelial-mesenchymal transition (EMT) is a tightly regulated and dynamic process occurring in both embryonic development and tumor progression. Our study aimed to comprehensively explore the molecular subtypes, immune landscape, and prognostic signature based on EMT-related genes in low-grade gliomas (LGG) in order to facilitate treatment decision-making and drug discovery.

**Methods:** We curated EMT-related genes and performed molecular subtyping with consensus clustering algorithm to determine EMT expression patterns in LGG. The infiltration level of diverse immune cell subsets was evaluated by implementing the single-sample gene set enrichment analysis (ssGSEA) and ESTIMATE algorithms. The distinctions in clinical characteristics, mutation landscape, and immune tumor microenvironment (TME) among the subtypes were subjected to further investigation. Gene Set Variation Analysis (GSVA) was performed to explore the biological pathways that were involved in subtypes. The chemo drug sensitivity and immunotherapy of subtypes were estimated through GDSC database and NTP algorithm. To detect EMT subtype-related prognostic gene modules, the analysis of weighted gene co-expression network (WGCNA) was performed. The LASSO algorithm was utilized to construct a prognostic risk model, and its efficacy was verified through an independent CGGA dataset. Finally, the expression of the hub genes from the prognostic model was evaluated through the single-cell dataset and *in-vitro* experiment.

**Results:** The TCGA-LGG dataset revealed the creation of two molecular subtypes that presented different prognoses, clinical implications, TME, mutation landscapes, chemotherapy, and immunotherapy. A three-gene signature (SLC39A1, CTSA and CLIC1) based on EMT expression pattern were established through WGCNA analysis. Low-risk patients showed a positive outlook, increased immune cell presence, and higher expression of immune checkpoint proteins. In addition, several promising drugs, including birinapant, fluvastatin, clofarabine, dasatinib, tanespimycin, TAK−733, GDC−0152, AZD8330, trametinib and ingenol-mebutate had great potential to the treatment of high risk patients. Finally, CTSA and CLIC1 were highly expressed in monocyte cell through single-cell RNA sequencing analysis.

**Conclusion:** Our research revealed non-negligible role of EMT in the TME diversity and complexity of LGG. A prognostic signature may contribute to the personalized treatment and prognostic determination.

## 1 Introduction

The efficacy of cancer chemotherapy and immunotherapy is often impeded by inter-patient and intra-tumor heterogeneity, as well as multifactorial drug resistance. Low-grade gliomas (LGGs), being a form of malignant brain tumor, are also confronted with the challenge of tumor heterogeneity and resistance to treatment (Weller et al., 2015). Even after undergoing standard surgical resection followed by radiotherapy and chemotherapy, individuals diagnosed with low-grade gliomas continue to have a bleak prognosis, with an average survival rate ranging from 2 to 10 years (Golub et al., 2019). Despite extensive efforts to improve clinical outcomes, more than half of LGG cases progress and evolve into therapy-resistant high-grade aggressive glioma over time (Claus et al., 2015). One of the primary objectives of precision medicine is to precisely identify patients who are likely to benefit from personalized treatment and to prescribe treatments that are tailored to the unique characteristics of their tumors, with the aim of achieving optimal therapeutic outcomes. Hence, in the realm of precision medicine, it is imperative to establish a more precise categorization of tumors to eradicate the heterogeneity and resistance to treatment associated with LGGs.

The process of epithelial-mesenchymal transition (EMT) is an essential differentiation program that is necessary for the development of tissues during embryogenesis. During this process, cells lose their epithelial characteristics and acquire mesenchymal migration properties (Yang et al., 2020). Aberrant activation of the EMT process is frequently observed in tumor proliferation, leading to the development of resistance towards conventional therapeutic interventions (Lambert et al., 2017; Shibue and Weinberg, 2017). EMT has the capability to enhance the potential of tumor cells for invasion and metastasis by facilitating their migration, disturbing cell-cell connections, disintegrating the basement membrane, and restructuring the extracellular matrix (ECM) (Miyoshi et al., 2004). Moreover, the process of EMT is linked with a higher percentage of cancer stem cells, reduced immune response against tumors and the development of resistance towards treatment (Akalay et al., 2013; Rhim et al., 2014), the study of therapeutic resistance from the perspective of EMT is one of cancer research focus. Thus, the presence of EMT, which is a hallmark of cancer, has been shown to be intimately linked with tumor invasion, metastasis, and the acquisition of chemotherapy resistance, all of which are critical biological processes in cancer development (van Staalduinen et al., 2018; Loret et al., 2019). Despite the existence of variations among tumor subtypes, the EMT process in LGG could potentially hold significant prognostic and molecular typing value. As a result of the highly hostile and intrusive characteristics of gliomas, it has been progressively acknowledged that the occurrence of EMT in gliomas may hold significant significance in the progression of glioma and the restructuring of the glioma microenvironment (Lu et al., 2012). Given the emergence of innovative immunotherapy techniques that offer groundbreaking cancer treatment alternatives, it is crucial to ascertain the immune profile of distinct EMT expression patterns, and their responsiveness to immunotherapy (Yamaguchi, 2016). In our study, while we have primarily relied on computational analyses of existing datasets, we have also performed experimental validation of the identified key genes. This approach underscores the critical role of EMT-related genes in the context of low-grade gliomas (LGG) and further highlights their significance in future research. By combining computational insights with experimental evidence, we aim to provide a more comprehensive understanding of the role of EMT in the expression, prognosis, immune tumor microenvironment (TME), clinical implications, and personalized treatment strategies for LGG.

## 2 Materials and methods

### 2.1 Data acquisition

**FIGURE 1:**
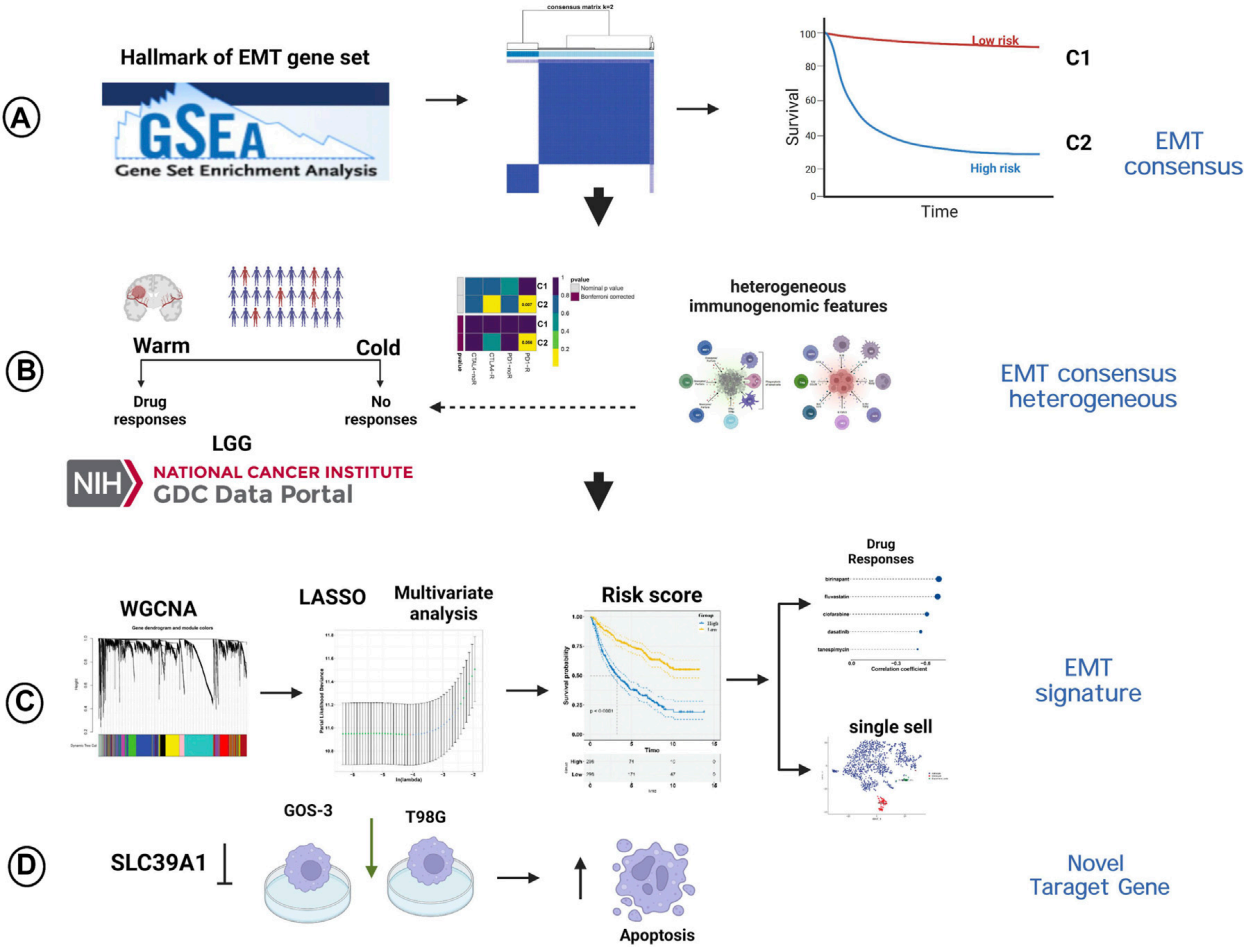
The graphical abstract and analysis flow chart of present study. **(A)** Construction of EMT-related consensus clusters. **(B)** Evaluation of immunotherapy of EMT-related consensus clusters. **(C)** Construction of EMT-related signature. **(D)** Experimental validation of SLC39A1.

Transcriptome data, mutation data and clinicopathologic characteristics of LGG samples were retrieved from the Cancer Genome Atlas (TCGA; https://www. cancer. gov/tcga/) through the Genomic Data Commons data portal (GDC; https://portal.gdc.cancer.gov/). In addition, the aforementioned information were obtained from two cohorts (mRNAseq_693, Illumina HiSeq Platform; mRNAseq_325, Illumina HiSeq (2000) and (2,500) platforms) in Chinese Glioma Genome Atlas (CGGA; http://www.cgga.org.cn), and serve as an external dataset. “SVA” (Leek et al., 2012) package was then used to merged the two cohorts after removing the batch effects (“ComBat” algorithm). After converting the raw read count, the values were expressed as transcripts per kilobase million (TPM). The Molecular signatures database (MSigDB; http://www.broadinstitute.org/msigdb) (Liberzon et al., 2015) provided a collection of 200 genes related to EMT, obtained from the gene set named “HALLMARK_EPITHELIAL_MESENCHYMAL_TRANSITION”. Figure 1 showed the graphical abstract and analysis flow chart of present study.

### 2.2 Unsupervised clustering analysis

To perform an unsupervised cluster analysis on mRNA expression profiles of genes associated with EMT, the “ConsensusClusterPlus” R package was created (Wilkerson and Hayes, 2010). The optimal number of clusters was determined using the cumulative distribution function (CDF) and consensus matrix through resampling analysis to achieve the best performance. To assess the disparity in survival among clusters, the Kaplan-Meier (KM) analysis was employed. The results displayed the top 20 mutated genes in each cluster after visualizing single-nucleotide polymorphisms (SNP) variations using the R package “maftools” (Mayakonda et al., 2018). The GISTIC2.0 tool from Genome Data Analysis Center (GDAC) Firehose (https://gdac.broadinstitute.org) was used to analyze the copy number alterations (CNA) data among subtypes.

### 2.3 Landscape of immune TME

To calculate the ratio of immune cells in LGG samples, the Estimation of Stromal and Immune cells in Malignant Tumours using Expression (ESTIMATE) data method was utilized to compute the stromal and immune scores (Yoshihara et al., 2013). The estimation of tumor purity was done by combining stromal and immune scores. The violin plot displayed variations in immune score, stromal score, and tumor purity across various subtypes.

The single-sample gene set enrichment analysis (ssGSEA) algorithm was utilized to measure the proportion of immune cell infiltration in the immune TME of LGG samples through the computation of enrichment scores. To compute the abundance of 28 tumor infiltrating immune cells (TIICs), a sum of 782 marker genes was collected from Bindea et al. (Bindea et al., 2013) and Broad Institute (http://software.broadinstitute.org/gsea/msigdb/index.jsp). Boxplots were used to present the results of ssGSEA analysis, which compared the level and function of immune infiltration among different subtypes using the gene set variation analysis (GSVA) software “GSVA” R package.

### 2.4 GSVA and functional annotation

To evaluate dissimilarities in biological processes among various clusters, the R software package “GSVA” was utilized for conducting GSVA analysis. Based on transcriptome data, GSVA is an unsupervised and nonparametric gene enrichment method that estimates alterations in the activity of biological processes and pathways in samples (Hänzelmann et al., 2013). To run GSVA analysis, the gene set of “C2. cp.kegg.v7.1″was downloaded from MSigDB database. The “clusterProfiler” R software package was utilized to conduct functional annotation of genes related to EMT. Significantly enriched pathways were identified through screening for those with an adjusted *p*-value < 0.05 and false discovery rate (FDR) < 0.05.

### 2.5 Therapy response prediction of subtype

The tumor immune dysfunction and exclusion (TIDE) algorithm (Jiang et al., 2018) was utilized to approximate the efficacy of immunotherapy based on tumor immune dysfunction and exclusion. According to the Genomics of Drug Sensitivity in Cancer (GDSC) database (https://www.cancerrxgene.org/) (Yang et al., 2012), we predict chemotherapy response of each LGG sample. After performing simple processing of data (such as elimination of low-variation genes and summarize duplicated gene expression data into averages), we estimated the sensitivity of eight chemotherapeutic agents (cisplatin, erlotinib, methotrexate, vincristine, carmustine, temozolomide (TMZ), rapamycin, and doxorubicin) currently under study or widely applied in gliomas by SubMap analysis (Gene Pattern) of GDSC data (Hoshida et al., 2007). The ridge regression was performed to estimate the half-maximal inhibitory concentration (IC50) for LGG by “pRRophetic” R software package (Geeleher et al., 2014a), and the quantitative prediction accuracy was verified by 10-fold cross validation based on the GDSC training set (Geeleher et al., 2014b).

### 2.6 Weighted correlation network analysis (WGCNA) to identify hub genes from subtypes

For the purpose of identifying subtype associated genes, we adopted WGCNA algorithm through the “WGCNA” R package (Version: 1.71) (Langfelder et al., 2009). At first, genes that exhibited a variance value exceeding 25% were selected for the construction of the coexpression network (Yang and Xu, 2021; Zhou et al., 2021; Feng et al., 2022). Subsequently, the outlier samples were excluded through the “goodSampleGenes” function, and the soft-thresholding value *β* = 5 (scale free = 0.85) was applied to ensure a scale-free network. Then, a gene clustering tree was generated based on the computed adjacency among genes, followed by the categorization of genes into distinct modules comprising no less than 100 genes exhibiting similar characteristics within each module. The modules that exhibited most correlation with subtypes were selected to the downstream analysis, such as KEGG analysis. Moreover, the hub genes were screened based on the cutoff gene significance (GS) > 0.6 and module membership (MM) > 0.8. Pathway enrichment analysis on the modules was performed via the “clusterProfiler” R package (Wu et al., 2021).

### 2.7 Development of EMT-related signature

The “glmnet” package was employed to conduct a LASSO regression analysis on genes associated with prognostic EMT subtyping to evaluate their effect on prognosis. The analysis was subjected to ten-fold cross-validation, and the genes were chosen based on the point with the lowest error rate. Patients were categorized into low or high EMT score subgroups using the median score, which was calculated by merging gene expression and coefficients to compute the EMT risk score. KM survival curves were generated by performing survival analysis with the aid of the “survival” and “survminer” packages. To generate receiver operating characteristic (ROC) curves the “timeROC” package was employed. Prognostic variables were subjected to uni- and multivariate-cox regression analyses. A nomogram was constructed using the “rms” package to estimate survival probability, and calibration curves were used to evaluate the accuracy of the predictions.

### 2.8 Drug discovery of EMT-related signature

The response of human cancer cell lines to small molecule compounds was estimated using drug sensitivity profiles obtained from either the Cancer Therapeutics Response Portal (CTRP) (Basu et al., 2013) or the PRISM project (Corsello et al., 2020).

### 2.9 Single cell RNA-sequencing (scRNA-seq) revealed the expression level of genes

The GSE202096 dataset provided scRNA-seq data for one LGG samples (GSM6094425) (Chen et al., 2022). After conducting quality control, cells that had more than 20% mitochondrial UMI counts were eliminated using the “Seurat” package (Cao et al., 2022). The selection process involved choosing the top 1,500 genes with high variability, followed by clustering cell populations via the FindClusters function and then mapping them into t-distributed stochastic neighbor embedding (t-SNE). Using the FindAllMarkers function, the markers for each cell cluster were determined. Then, the CellMarker database was utilized to annotate cells based on their cell markers (Hu et al., 2022).

### 2.10 Cell culture and transfection

The cultivation of HMC3, GOS-3, T98G, and LN-18 cells (ATCC) was carried out in DMEM supplemented with 10% fetal bovine serum (Sigma Aldrich) under an atmosphere of 5% CO2 at 37°C. After cloning siRNA targeting SLC39A1 into lentiviral vectors, the vectors were transfected into GOS-3 and T98G cells for 3 days and then exposed to 4 μg/mL puromycin for 1 week.

### 2.11 Real-time quantitative PCR (RT-qPCR)

Total RNA was extracted using TRIzol (Beyotime) and cDNA was synthesized with the PrimeScript RT reagent kit after gDNA Eraser (Takara) treatment. SYBR Green II Mixture (TaKaRa) was used for RT-qPCR, and GAPDH was used as an internal reference to calculate the expression using the 2^−ΔΔCT^ method.

### 2.12 Flow cytometry

Cell apoptosis was detected through flow cytometry and the Cell Apoptosis Detection Kit (KTA0002; Abbkine) was utilized. To summarize, a group of cells was exposed to 5 μL of Annexin V-AbFluor™ 488 binding and 2 μL of PI for 15 min at room temperature while being shielded from light. After adding 400 μL of 1 x Annexin V buffer, the level of apoptosis was assessed using a Beckman Flow cytometer.

### 2.13 Statistical analysis

The R language (R-project.org) platform and software package from the Bioconductor project (www.bioconductor.org) (R Core Team, Version 4.0.2) were used for statistical analysis and visualization of results in the current study. To compare the two groups and more groups, the Wilcoxon and Kruskal–Wallis tests were utilized, respectively (Hazra and Gogtay, 2016). To detect the differentially expressed genes (DEGs) between the two subtypes, the one-way ANOVA and Tukey’s test were conducted with a q-value of less than 0.05 and an absolute value of log2FC greater than 2. The KM curve, analyzed by log-rank test, exhibited the variations in operating systems among different groups. Bilateral *p* values were used, with a statistically significant difference defined as *p* < 0.05.

## 3 Results

### 3.1 Clinicopathological characteristics of two LGG molecular subtypes based on EMT related genes

**FIGURE 2:**
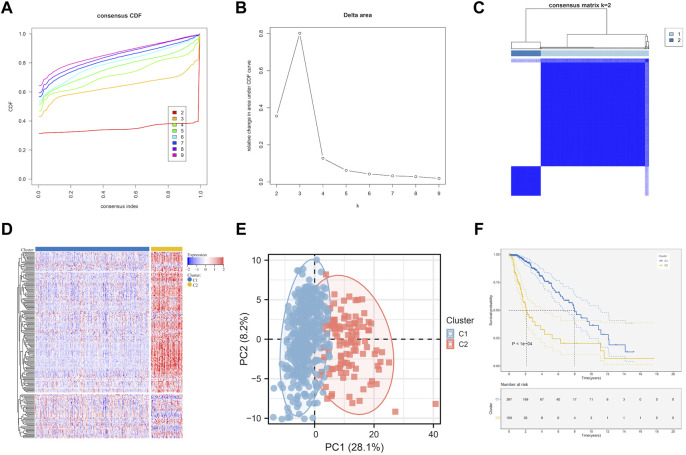
EMT-related genes could distinguish LGG in TCGA with different clinical and molecular features. **(A)** Relative change in area under CDF curve for k = 2 to k = 9. **(B)** Delta curve analysis from k = 2 to k = 9. **(C)** Consensus clustering matrix heatmap plots of 506 samples from TCGA datasets for k = 2. **(D)** Expression of EMT-related genes in molecular subtypes. **(E)** PCA analysis of the EMT-related genes expression when k = 2. **(F)** Kaplan-Meier analysis of patients between two subtypes. CDF, cumulative distribution function; PCA, principal components analysis.

Based on the prognostic EMT gene expression profile, LGG samples was divided into two molecular subtypes by unsupervised clustering analysis (Figures 2A–C). The EMT gene expression were presented a significant divergence between two subtypes (Figure 2D). Figure 2E showed that the principal components analysis (PCA) distribution patterns were mostly in agreement with the two subtypes designations. Figure 2F suggested the overall prognosis of C2 is worse than that of C1 (*p* < 0.0001). Moreover, we further explore the relationship between subtypes and clinical traits.

**FIGURE 3:**
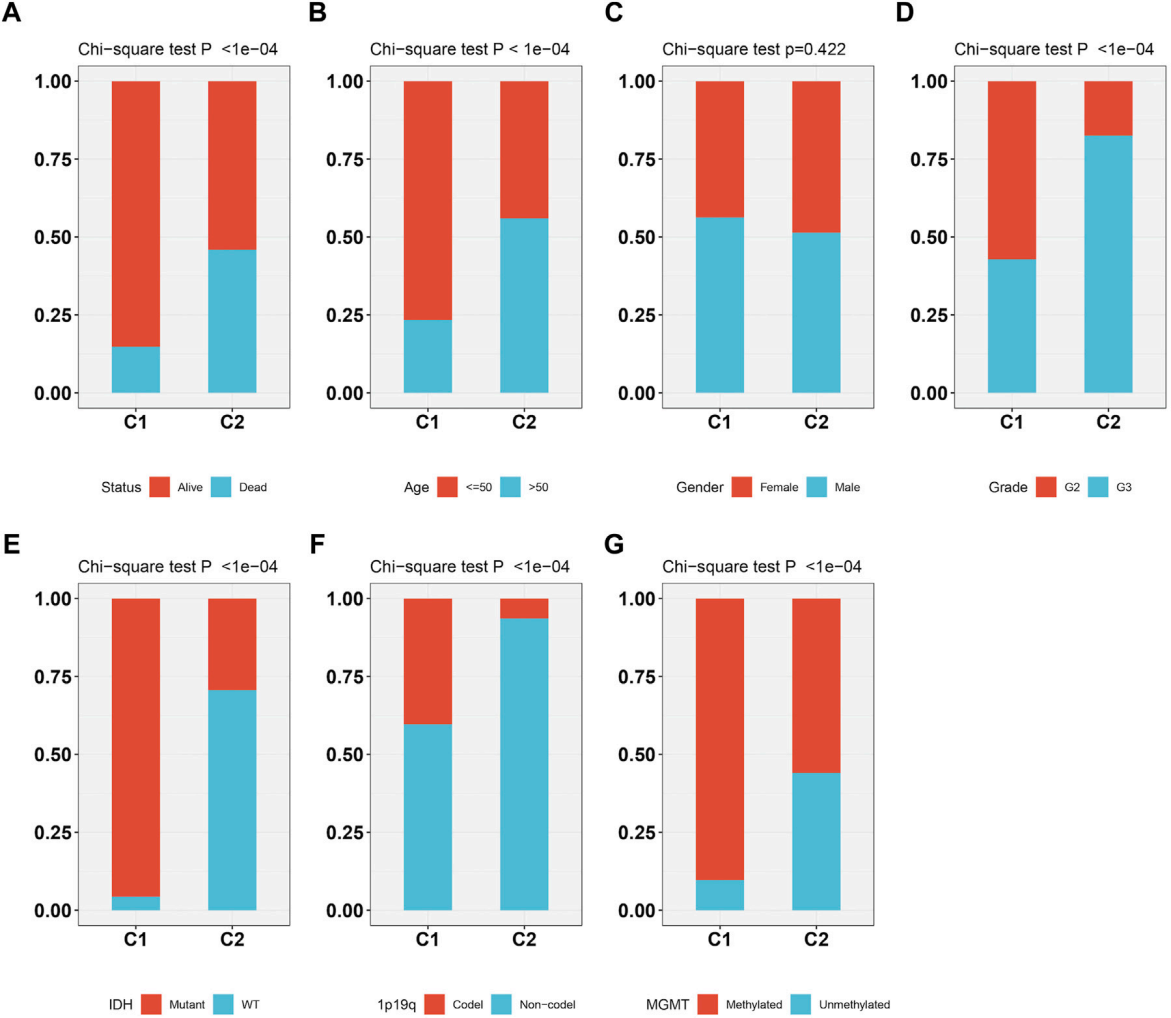
The relationship between two molecular subtypes, survival status **(A)**, age **(B)**, gender **(C)**, grade **(D)**, IDH mutation status **(E)**, 1p19q status **(F)** and methylation status **(G)**.

As Figures 3A–G showed, the patients survival status (Figure 3A), age (Figure 3B), grade (Figure 3D), IDH status (Figure 3E), 1p19q status (Figure 3F) and methylation status (Figure 3G) were showed a significant difference (*p* < 0.05) between C1 and C2, while no difference was observed in gender (Figure 3C). Subtype C2 corresponding to more deaths, elderly patients, higher grade, 1p19q non-codel, IDH wild type, and unmethylated cases.

We assessed the function of each subtype through GSVA analysis (Supplementary Figure S1) to delve deeper into the potential biological process of the two clusters. Results suggested that C1 mainly enriched metabolism-related signaling pathways, such as butanoate metabolism and propanoate metabolism pathways. The primary routes of C2 that were enriched consist of cell adhesion molecules (CAMs), interaction with ECM receptors, the p53 signaling pathway, interaction between cytokines and cytokine receptors, focal adhesion, the JAK-stat signaling pathway, and apoptosis. These pathways were significantly correlated with tumor cell genesis, proliferation, invasion and migration. Furthermore, the GSVA outcomes indicated that C2 was linked to pathways related to DNA damage repair (such as Nucleotide excision repair, DNA replication, and Mismatch_repair) as well as immune functions (including Natural killer cell mediated cytotoxicity, Toll like receptor signaling pathway, and Antigen processing and presentation). In conclusion, C2 showed more malignant biological behaviors and enrichment signaling pathways than C1.In addition, SNP analysis of the two clusters in TCGA uncovered that C1 had a higher mutation proportion than C2 of IDH1, TP53, ATRX, CIC, FUBP1, NOTCH1 (Rotin et al., 2015) and IDH2 (Supplementary Figure S1A). However, EGFR, PTEN and NF1 (Verhaak et al., 2010; Marques et al., 2019; Koptyra et al., 2023), the mutations that were common in glioblastoma (GBM), were more frequent in C2 than C1 (Supplementary Figure S2B). Therefore, EMT related genes are important references for LGG molecular subtype. Our cluster-specific CNAs analysis presented chromosome deletions and amplifications, for example, deletion of 9p21.3 were significantly enriched in the C2. The 9p21.3 deletions is associated with the poor survival outcome in LGG (Xia et al., 2021). In addition, gene duplication/amplification at 7p11.2 activates EGFR expression through the formation of new topological associating domain (TAD) and the emergence of new enhancer-promoter interactions between LINC01446 and EGFR, and EGFR amplification and/or mutations are directly associated with poor prognosis of gliomas (Yang et al., 2022).

### 3.2 Disparity in immune tumor microenvironment between subtypes

**FIGURE 4:**
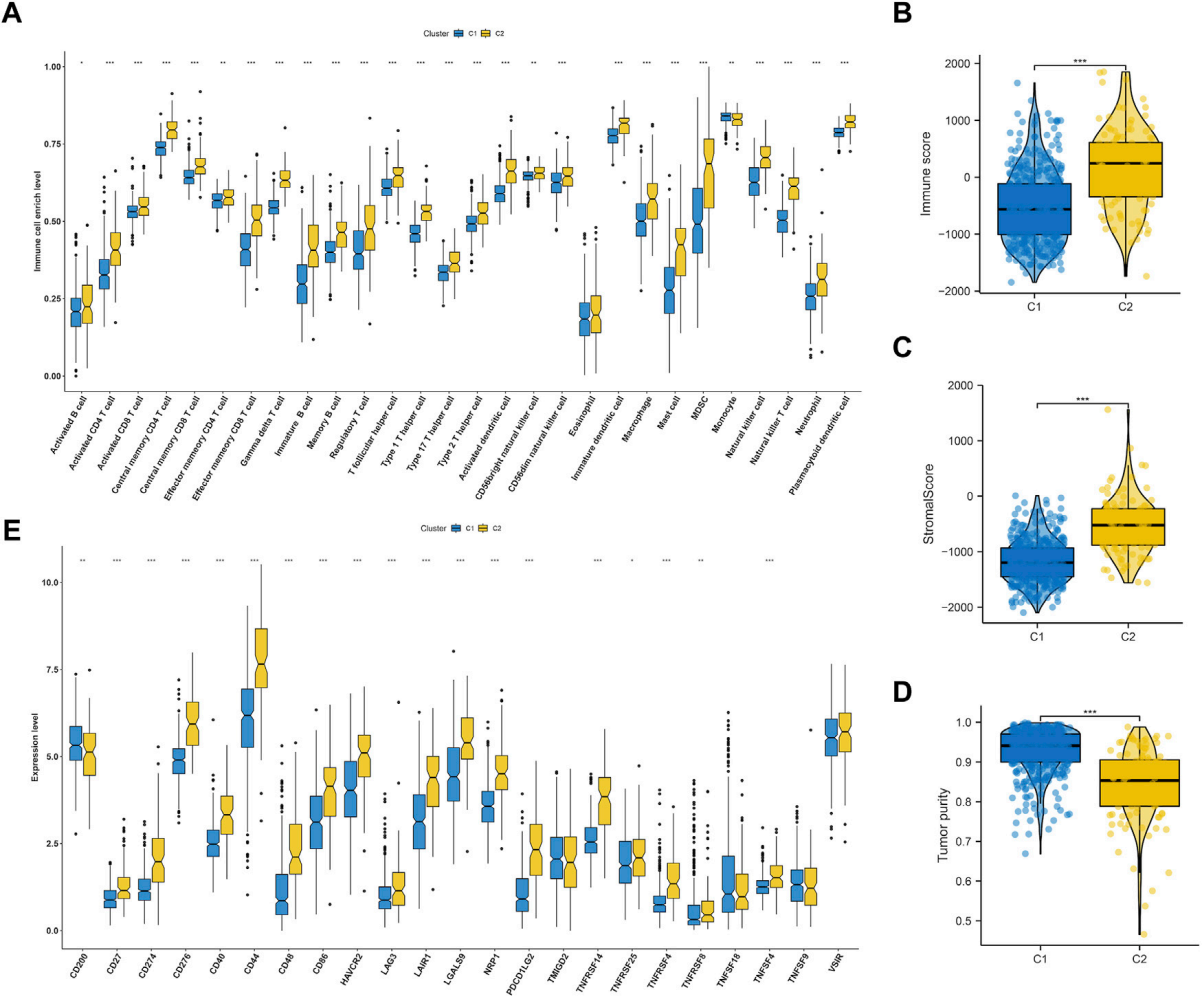
Immune landscape of subtypes in LGG. **(A)** Comparisons of 28 immune cells infiltration level in two molecular subtypes. Evaluation of the immune score **(B)**, stromal score **(C)** and tumor purity **(D)** in two molecular subtypes. **(E)** Comparisons of key immune checkpoints expression level in two molecular subtypes.

We delved deeper into the dissimilarities in the immune tumor microenvironment between the two LGG EMT subtypes, given their notable clinicopathologic distinctions. According to Figure 4A, C2 had a higher degree of immune cell infiltration than C1, which included various types of immune cells such as Activated CD8^+^ T cells, effector memory CD4^+^ T cells, Regulatory T cells, CD56bright natural Killer cells, Type-1 T-helper 1 cells, Central memory CD4^+^ T cells, Activated Dendritic cells, Activated CD4^+^ T cells, CD56dim natural killer cells, myeloid-derived suppressor cells, Immature Dendritic cells, Central memory CD8^+^ T cells, Effector memory CD8^+^ T cells, immature B cells, macrophages, mast cells, memory B cells, natural killer cells, natural killer T cells, neutrophils, plasmacytoid dendritic cells, follicular helper T cells, Gamma delta T cells, Type-17 T helper cells, Type-2 T helper cells, activated B cells, eosinophils and monocytes. The findings from analyzing various cluster immune TME in TCGA and CGGA datasets indicated a strong correlation between the EMT molecular subtype and immune TME. It was noticed that C2 had higher stromal and immune scores compared to C1 (both *p* < 0.0001) as shown in Figures 4B,C. On the other hand, the tumor purity in C2 was lower than that in C1 (*p* < 0.0001) as depicted in Figure 4D. Figure 4E showed that C2 had significantly higher expression levels of most checkpoints compared to C1.

### 3.3 Sensitivity prediction of different clusters to chemotherapy and immunotherapy

**FIGURE 5:**
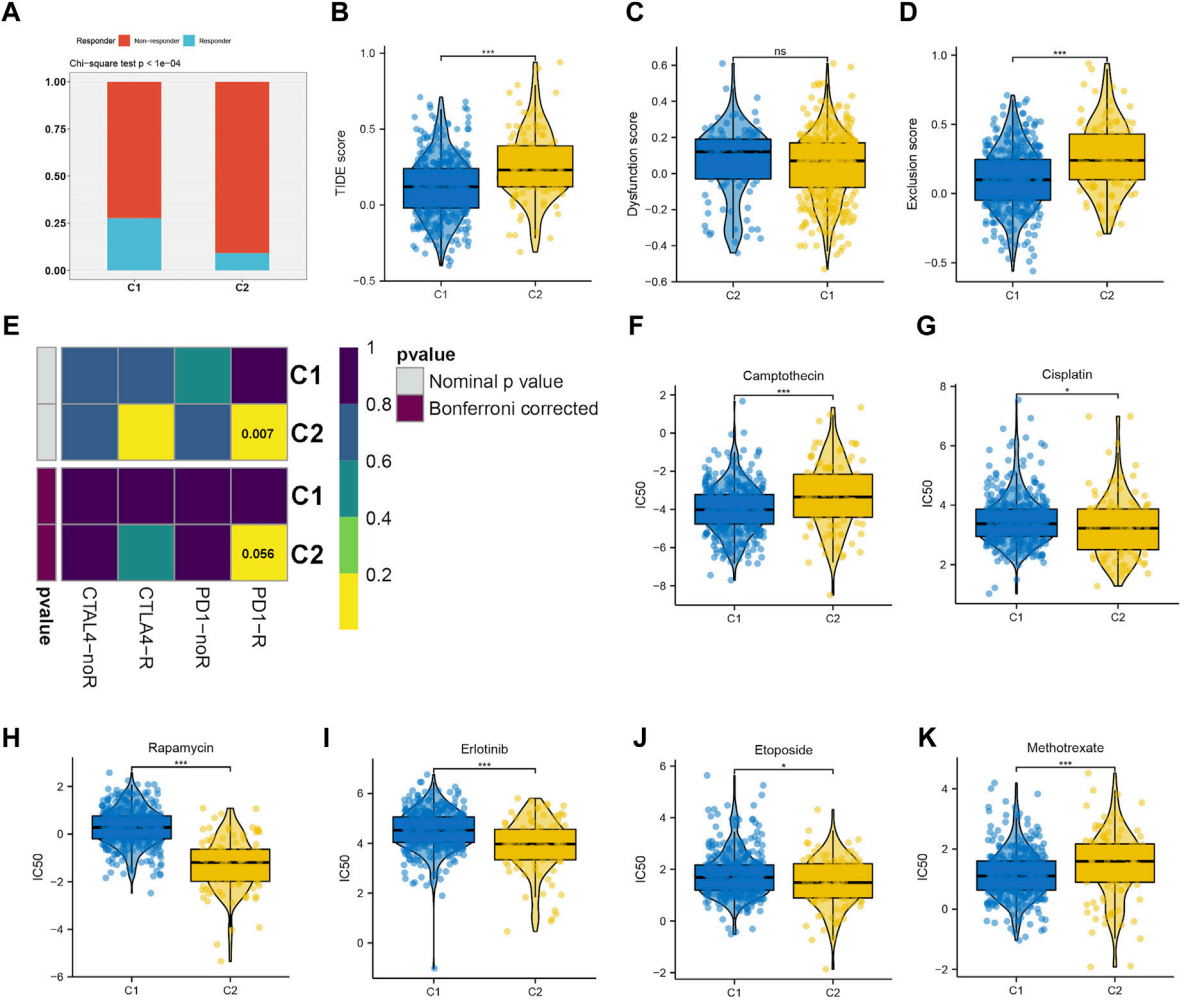
**(A–D)** The immune response, TIDE score, dysfunction score and exclusion score of the C1 and C2 subtype. **(E)** Submap analysis revealing that C2 would be more sensitive to immunotherapy, especially checkpoints PD-1 immunotherapy (Bonferroni-corrected *p* < 0.05). Drug sensitivity evaluation of the Camptothecin **(F)**, Cisplatin **(G)**, Rapamycin **(H)**, Erlotinib **(I)**, Etoposide **(J)**, Methotrexate **(K)** in C1 and C2 subtypes.

Immunotherapy has shown a potential application prospect in the treatment of glioma. We evaluated the response of two clusters to immunotherapy through TIDE analysis. The findings revealed that patients belonging to C1 exhibit a higher number of responders in comparison to C2 (*p* < 0.05) (Figure 5A), and individuals with a lower TIDE score are more likely to experience benefits from immunotherapy. The TIDE, Dysfunction, and Exclusion scores in the C1 group were lower, which implies that they could benefit more from immunotherapy (Figures 5B–D). Immune checkpoint blockade (ICB) is one of the most promising therapeutic approaches to change the current pessimistic situation in the treatment of glioma. Therefore, SubMap module analysis was used to compare the expression profile of LGG cluster with other published ICB-therapy datasets. As showed in Figure 5E, Subtype C2 is similar to the melanoma patients who reacted positively to anti-PD-1 therapy.

We then evaluated the different responses of two clusters to drug chemotherapy. The regimen of combined treatment for gliomas includes maximum safe surgical resection and postoperative chemotherapy with radiotherapy. Glioma can be treated with chemotherapy, which is considered a fundamental therapy. The GDSC database was utilized to predict the efficacy of 8 frequently used drugs in LGG chemotherapy, including cisplatin, erlotinib, methotrexate, camptothecin, etoposide, rapamycin, and doxorubicin. According to the findings, camptothecin (Figure 5F) and methotrexate (Figure 5K) were found to be more effective on C1, whereas cisplatin (Figure 5G), rapamycin (Figure 5H), erlotinib (Figure 5I), and etoposide (Figure 5J) were more effective on C2.

**FIGURE 6:**
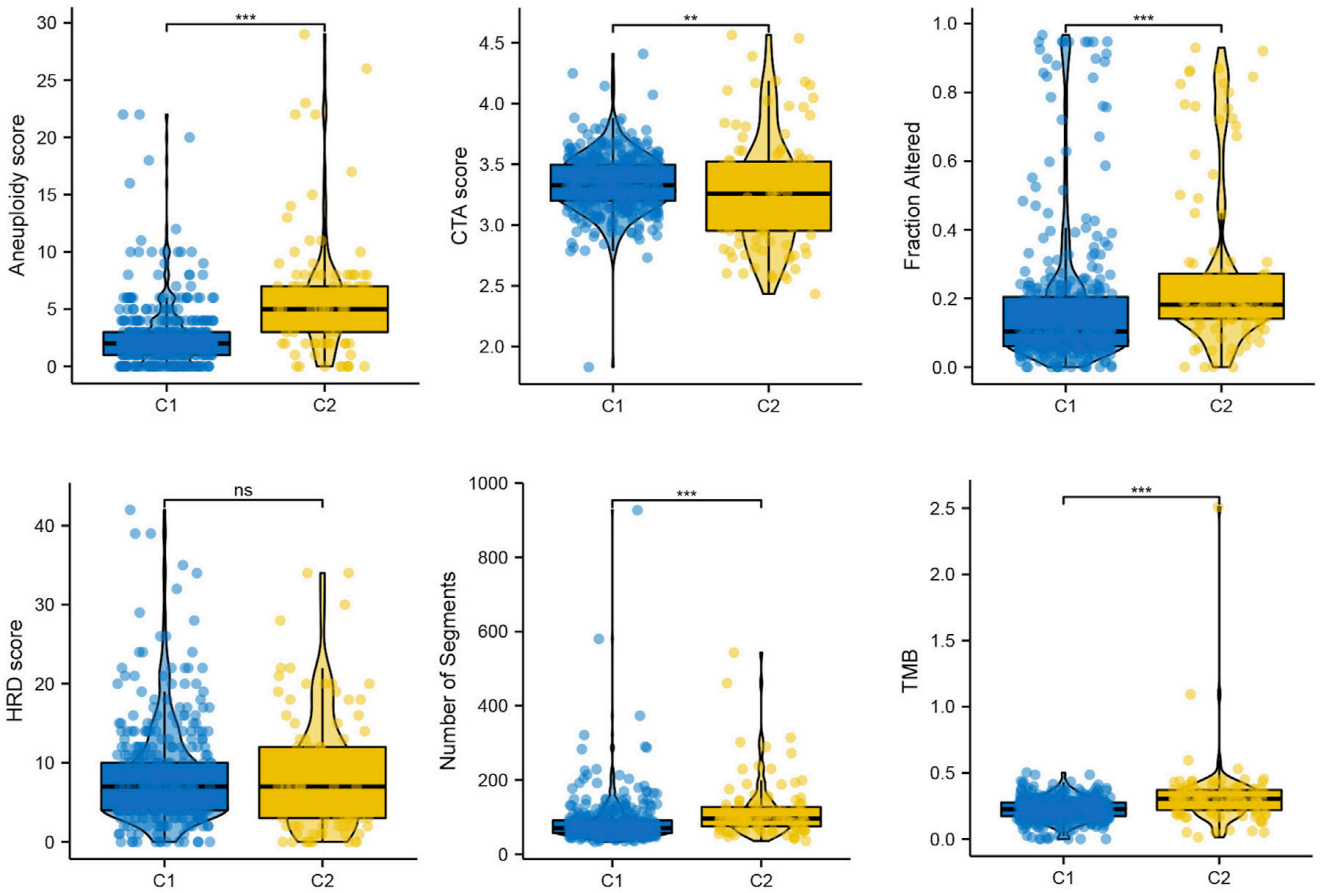
The mutation profile in subtypes, including Aneuploidy score, CTA score, Fraction altered, HRD score, Number of segments, and TMB.

Genomic instability is a hallmark of cancer, particularly LGG, which facilitates its growth and the ability to withstand treatments (Dionellis et al., 2021). In our result, we found that Aneuploidy score, fraction altered, number of segments, tumor mutation burden (TMB) were elevated in C2, while Cancer Testicular Antigens (CTA) score were lower in C2, which consistent with previously reports (Nie et al., 2020) (Figure 6).

### 3.4 Identification of EMT-related genes through WGCNA analysis

**FIGURE 7:**
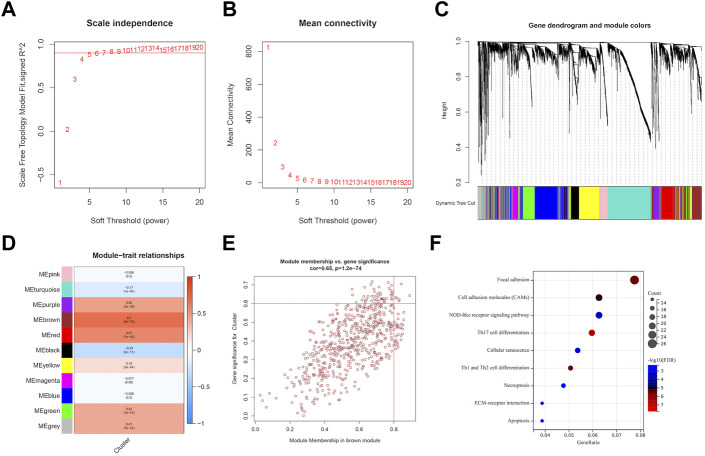
Identification of EMT-related genes through WGCNA analysis. **(A, B)** soft threshold determination through scale-free network analysis. **(C)** Clustering dendrograms were used to classify genes with similar expression patterns in LGG into co-expression modules. **(D)** module-traits heatmap showed the correlation between Cluster and gene modules. **(E)** hub genes were characterized by brown module with module membership >0.8 and gene significance >0.6. **(F)** Pathway enrichment analysis.

We utilized WGCNA to build correlation networks in order to identify genes associated with EMT. The optimal soft threshold was set to 5, based on a chosen R2 of 0.90 (Figures 7A,B). Eleven modules were identified based on their connectivity in Figure 7C, each containing at least 100 genes. The “module-trait” relationship heatmap revealed that brown module have the highest correlation associated with molecular subtypes (R = 0.70) (Figure 7D). In addition, six hub genes were identified, namely, SLC39A1, CTSA, TMSB4X, CAST, S100A11, and Chloride Intracellular Channel 1 (CLIC1), based on their GS being greater than 0.6 and their MM being greater than 0.8, as shown in Figure 7E. Further function enrichment of the brown module indicated that genes may involve in focal adhesion, Th17 cell differentiation, necroptosis, etc (Figure 7F).

**FIGURE 8:**
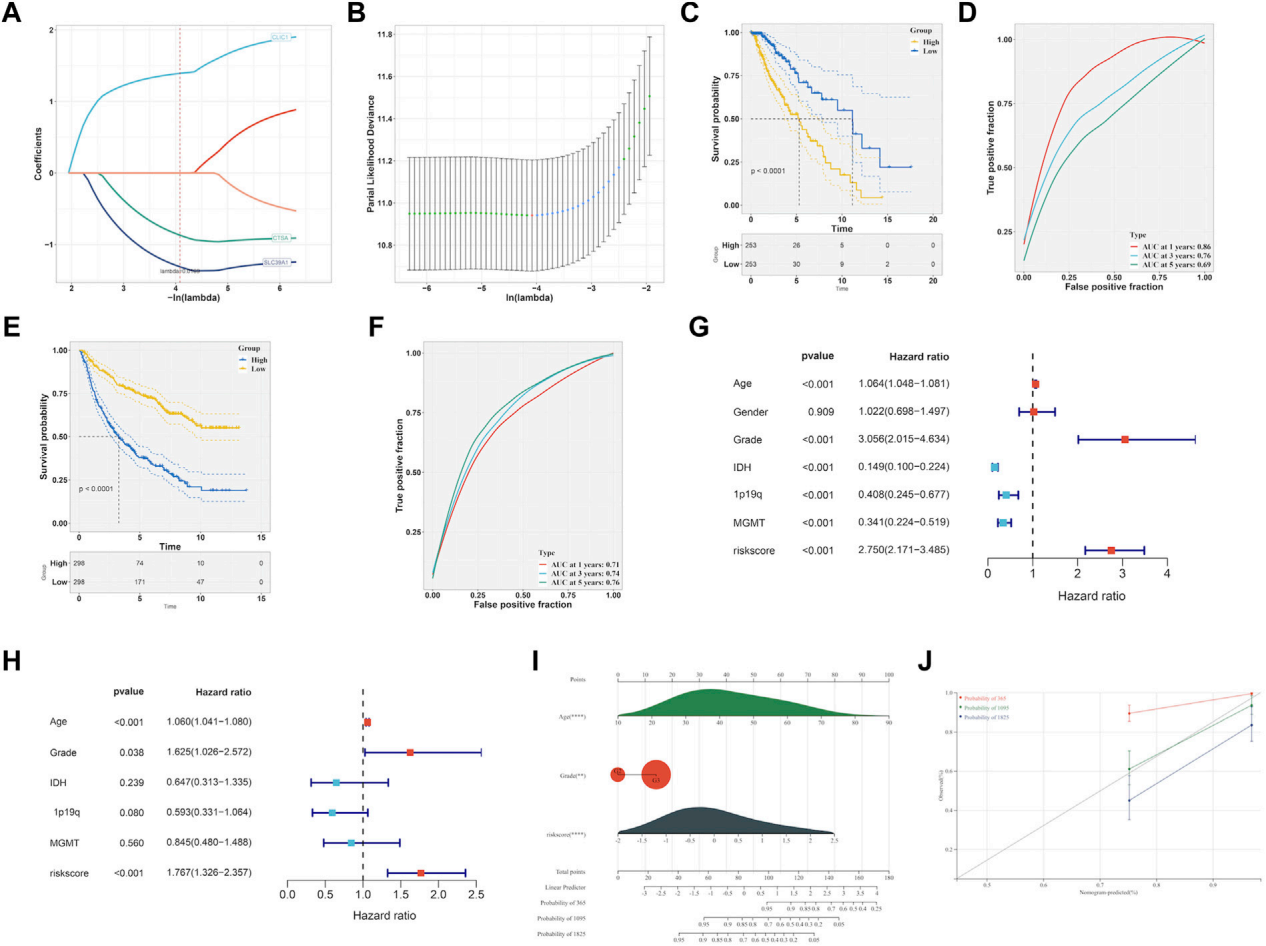
Development of EMT-related signature. **(A, B)** LASSO analysis to determine the EMT-related signature. **(C)** K-M curve survival analysis between high- and low-risk group in TCGA-LGG dataset. **(D)** ROC curve analysis of the EMT-related signature in TCGA-LGG dataset. **(E)** K-M curve survival analysis between high- and low-risk group in CGGA dataset. **(F)** ROC curve analysis of the of EMT-related signature in CGGA dataset. Determination of the independence of Univariate cox regression **(G)** and multivariate cox regression **(H)** analysis of the EMT-related signature. **(I)** Construction of nomogram on the basis of independent prognostic factors. **(J)** Calibration plot for the nomogram in 1-year, 3-year and 5-year OS.

To identify prognostic genes related to EMT from six hub genes, we performed lasso analysis and found three genes, namely, CLIC1, CTSA, and SLC39A1, based on the optimal lambda value (Figures 8A,B). The CGGA dataset was used as an external validation cohort to guarantee its reliability. The score for the risk model was computed utilizing the risk equation Risk score = (SLC39A1exp * −1.559) + (CTSAexp* −1.139) + (CLIC1exp * 1.474). After analyzing the TCGA and CGGA datasets, patients were divided into two groups: high-risk and low-risk, with the median score serving as the differentiating factor for each group. Figure 8C and Figure 8E demonstrated that patients who had a high-risk score in both TCGA and CGGA datasets had a poor survival outcome, whereas those with a low risk score had a favorable survival. Furthermore, the precision of the risk model was assessed using ROC curve analysis, yielding a score of 0.86, 0.76, and 0.69 for 1-, 3-, and 5-year periods in the TCGA and CGGA datasets, as shown in Figure 8D and 0.71, 0.74, and 0.76 in Figure 8F, respectively. The independent prognostic factors were identified through the results of both univariate and multivariate Cox regression analyses, demonstrating that the risk score can function in this manner, as depicted in Figures 8G,H. Then, the independent factors including risk score, grade were including to construct nomogram to predict patients overall survival (OS) (Figure 8I). According to Figure 8J, the calibration curves indicated that the estimated OS values were consistent with the real values, particularly for the 3-year OS.

### 3.5 Immune infiltration landscape and drug discovery of EMT-related model

**FIGURE 9:**
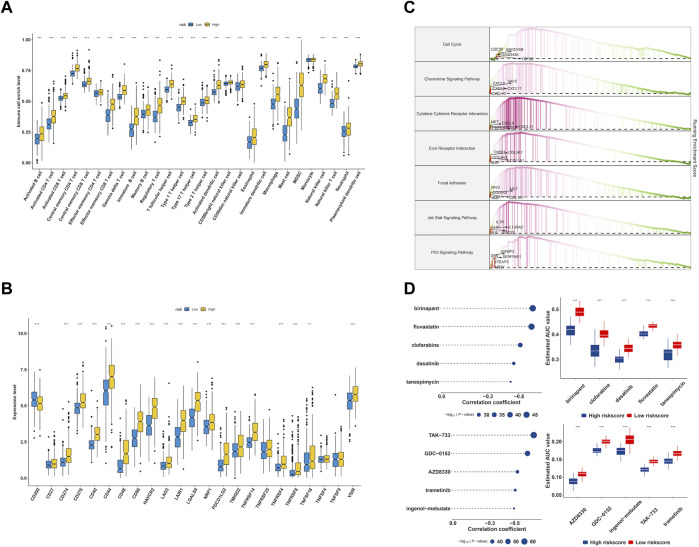
Characterization of immune infiltration and drug discovery of EMT-related signature. **(A)** Evaluation of 28 immune cells infiltration in high- and low-risk group. **(B)** The expression level of immune checkpoints in high- and low-risk group. **(C)** GSEA enrichment analysis of EMT signature. **(D)** Identification of molecular compounds in the high-risk groups based on CTRP and PRISM drug database.

Though the immune cell infiltration analysis, we evaluated the divergence of immune cells between risk groups. The result of Figure 9A revealed that most of immune cells infiltration level in high risk group were elevated compared to low risk group, such as Activated CD8^+^ T cells (CD8^+^ Ta), effector memory CD4^+^ T cells, Regulatory T cells, CD56bright natural Killer cells, Type-1 T-helper 1 cells, Central memory CD4^+^ T cells, Activated Dendritic cells, Activated CD4^+^ T cells, CD56dim natural killer cells, myeloid-derived suppressor cells, Immature Dendritic cells, Central memory CD8^+^ T cells, Effector memory CD8^+^ T cells, immature B cells, macrophages, mast cells, memory B cells, natural killer cells, natural killer T cells, neutrophils, plasmacytoid dendritic cells, follicular helper T cells, Gamma delta T cells, Type-17 T helper cells, Type-2 T helper cells, activated B cells, eosinophils and monocytes. We further checked the expression of immune checkpoint between groups and found that the genes showed a high expression level in high risk group (Figure 9B). Accumulative evidence have demonstrated that anti-tumor effects of a high T cell infiltration are counteracted by the immunosuppressive pathways that are triggered by the over-expression of immune checkpoint proteins (Vuong et al., 2019; Dionellis et al., 2021). The GSEA analysis was performed between groups, and found cell cycle, chemokine signaling pathway, cytokine-cytokine receptor interaction, ECM receptor interaction, p53 signaling pathway were significantly enriched in high risk group (Figure 9C).

To explore potential molecular drugs to the treatment of LGG in high risk group, we integrated CTRP and PRISM drug database. As showed in Figure 9D, the top panel of CTRP result revealed five drugs including birinapant, fluvastatin, clofarabine, dasatinib and tanespimycin may have the potential value to the treatment of high risk LGG patients, and the bottom panel of PRISM results indicated five drugs including TAK−733, GDC−0152, AZD8330, trametinib and ingenol−mebutate were identified.

### 3.6 Evaluation of gene expression level through single cell analysis

**FIGURE 10:**
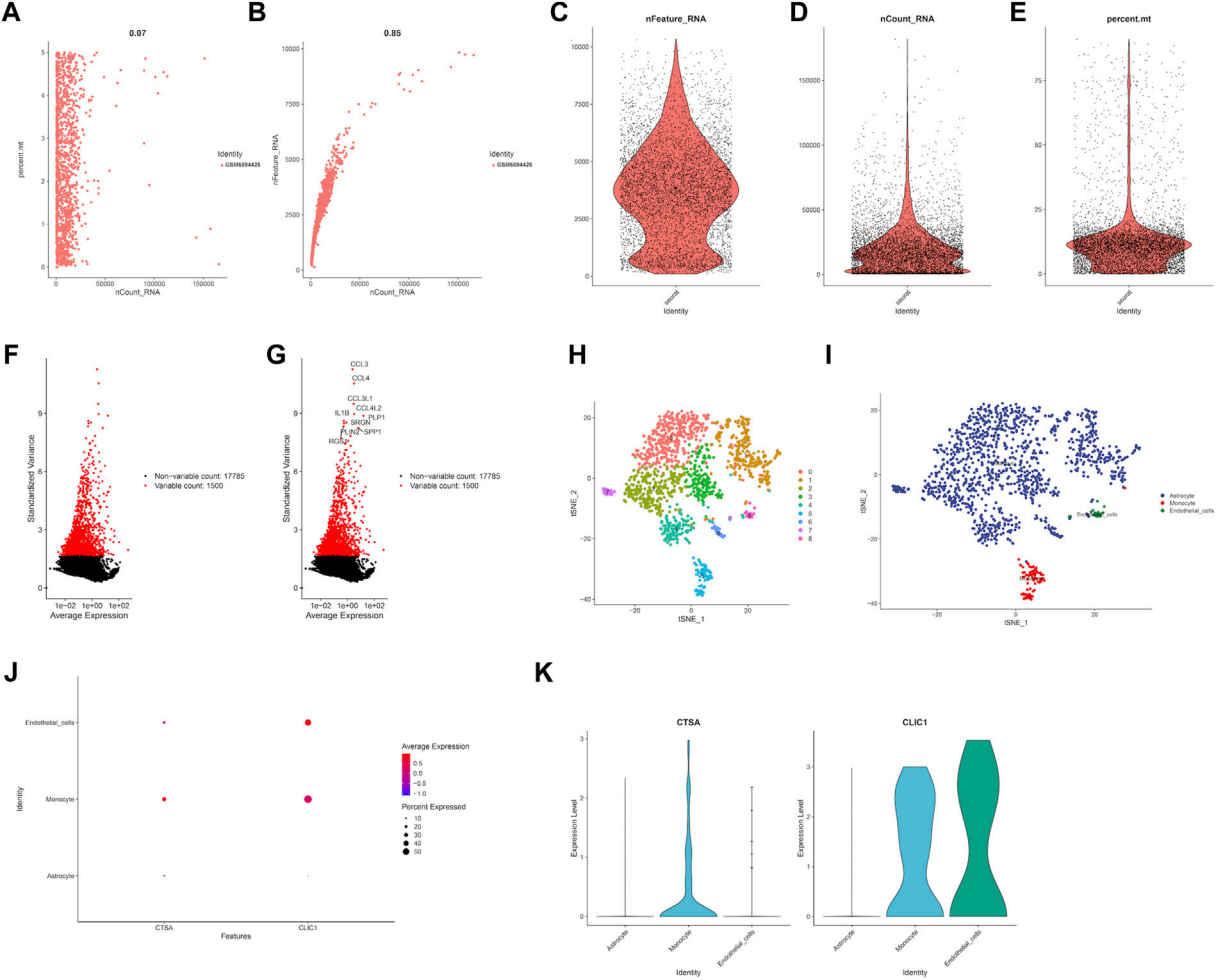
Single cell RNA analysis of LGG samples. **(A)** Evaluation of relationship between percentage of mitochondrial genes and mRNA readings. **(B)** Evaluation of relationship between percentage of mRNA quantity and mRNA readings. **(C–E)** A scatter plot illustrates the quantity of genes, UMI, and the percentage of mitochondrial genes in each cell type from the single LGG sample before quality control. **(F–G)** The red dots show the 1,500 genes that have the highest variability. **(H)** tSNE plot showed the cell population of LGG samples. **(I)** Cell annotation using cell markers for each cell. Expression level of prognostic genes in each cell type through dot plot **(J)** and violine plot **(K)**.

In order to estimate the gene expression level of hub genes, we retrieved LGG single cell sample from GSE202096 dataset (Figures 10A–E). Following data filtering and standardization, 1,500 genes exhibiting the most variance were selected for cell classification (Figures 10F,G). Dimensionality reduction of the expression levels of 1,500 genes was done by PCA analysis, resulting in PC1 to PC20. T-SNE was then applied to the PC1-20 to classify all cells into 9 clusters (Figure 10H). Afterwards, we annotated the cells in each cluster. The main cell types identified were astrocyte, monocyte, and endothelial cells (Figure 10I). The expression level of hub genes (CTSA, CLIC1) in cell clusters were showed in Figures 10J,K, of which CTSA was highly expressed in monocyte, and CLIC1 highly expressed in monocyte and endothelial cells.

### 3.7 Experiment validation of the hub genes

**FIGURE 11:**
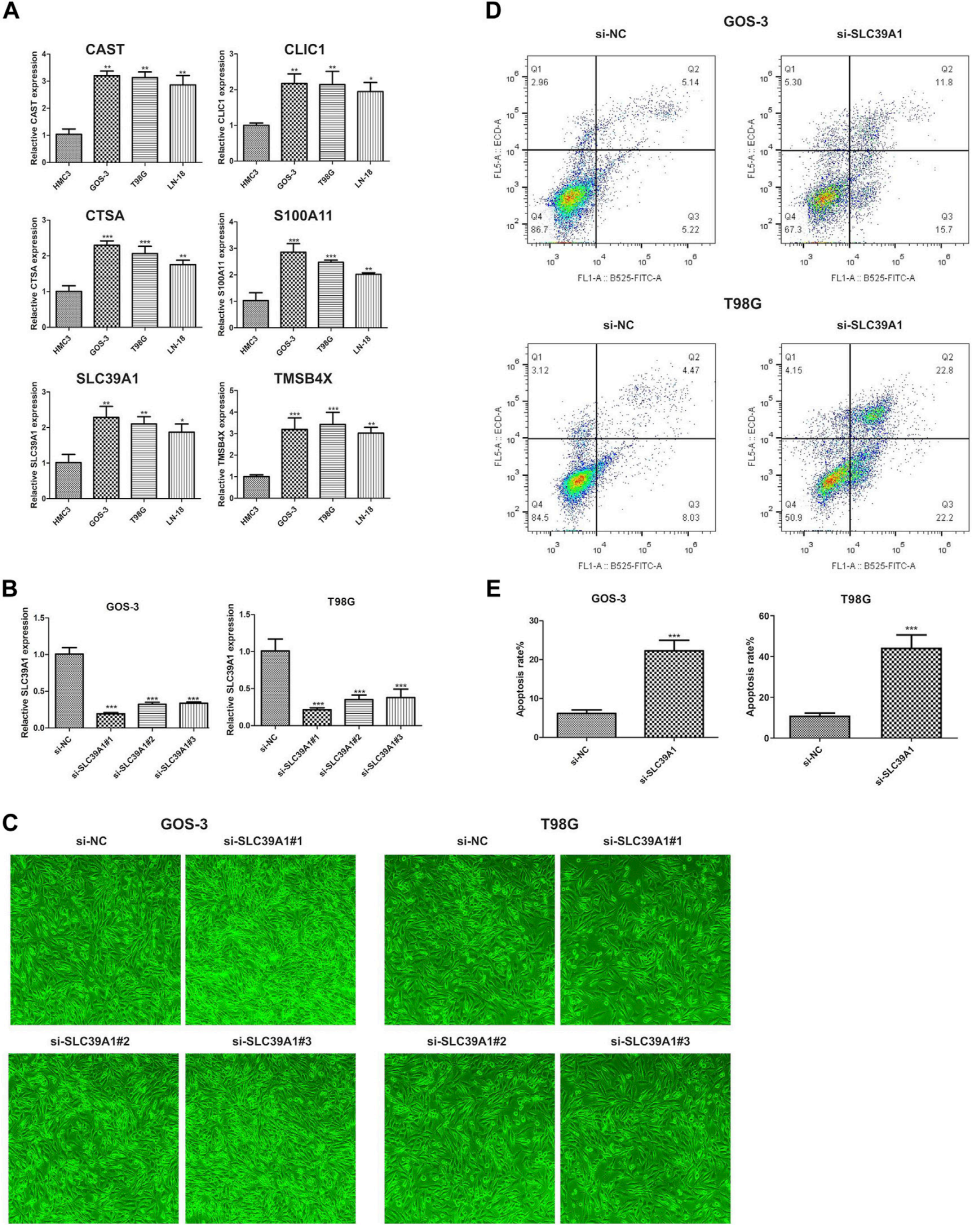
Validation of hub gene through *in-vitro* experiment. **(A)** Six hub genes expression level in HMC3, GOS-3, T98G, LN-18 cell lines. **(B)** SLC39A1 expression level in GOS-3 and T98G cell lines with SLC39A1 silence. **(C)** Transfection of si-SLC39A1 was filtered with puromycin for 1 week, and the expression of si-NC and si-SLC39A1 was observed by light microscopy. **(D, E)** Apoptotic level of SLC39A1-knockout GOS-3 and T98G cells.

As depicted in the previously result, we obtained six hub genes (CAST, CLIC1, CTSA, S100A1, SLC39A1 and TMSB4X) associated with EMT from WGCNA result. Next, we investigated the level of expression of the six genes in four LGG cell lines, namely, HMC3, GOS-3, T98G, and LN-18. Figure 11A demonstrates that the majority of genes were expressed at high levels in both COS-3 and T98G cell lines, as indicated by the RT-PCR results. Using the gene expression profiling interactive analysis (GEPIA) database, we delved deeper into the expression levels of normal and tumor tissue. Our findings revealed that CLIC1, S100A1, and SLC39A1 were significantly highly expressed in tumor tissue compared to normal tissue. However, CAST, CTSA, and TMSB4X showed no significant difference (Supplementary Figure S3). Prior research indicated that CLIC1 and S100A1 are involved in the advancement of LGG, thus our focus shifted to examining the function of SLC39A1 in LGG. Firstly, we examined the expression level through the RT-PCR experiment. Two LGG cell lines, GOS-3 and T98G, were treated with si-SLC39A1 #one to three, resulting in a notable reduction in SLC39A1 expression as determined by RT-qPCR analysis (Figure 11B). Meanwhile, we screened the stably transfected cell lines with puromycin after transfection of si-SLC39A1 #one to three, and the construction of stably transfected cell lines was observed by light microscopy 1 week after screening. The results showed that the stable-transformed cell line of si-SLC39A1 was successfully constructed by puromycin screening (Figure 11C). Flow cytometry was conducted to assess the apoptosis of GOS-3 and T98G cells, by comparing the number of cells distributed in early apoptosis and late apoptosis, the results showed that the apoptotic cell number in Q2 and Q3 regions of the si-SLC39A1 group was much higher than that of the si-NC group in both GOS-3 and T98G cell lines, revealing that the apoptosis rate was greater in cells with SLC39A1 knockout, indicating that the reduction of SLC39A1 significantly induced apoptosis of LGG cells (Figures 11D,E).

## 4 Discussion

In the present study, a comprehensive analysis of LGG in TCGA and CGGA datasets was conducted based on EMT-related genes. By data mining analysis, we identified two clusters with markedly different prognosis and clinical phenotype in LGG. Further analysis showed that the two clusters were significantly different in TME immune cell infiltration and functional pathways, and presented inconsistent sensitivity to chemotherapy and immunotherapy. Finally, we developed a EMT-related signature and screen small molecule compounds with potential therapeutic effect targeting EMT. Additionally, we have undertaken further experimental validation of the identified EMT-related genes and their role in LGG. To some extent, our research contributed to improving the current LGG treatment dilemma in precision therapy such as molecular classification, drug development, chemotherapy and immunotherapy.

The ESTIMATE and ssGSEA algorithms were used to measure the number of immune cells and stromal cells in every LGG sample from the TCGA and CGGA datasets. According to our data, immune and stromal scores as well as tumor purity vary among various LGG-EMT subtypes. Furthermore, our findings indicate that C2, which represents the LGG-EMT subtype with unfavorable prognosis, is associated with a greater level of infiltration of various immune cells such as CD56bright and CD56dim natural killer cells, eosinophils, monocytes, plasmacytoid dendritic cells, and others. Monocytes, which are believed to have tumor-promoting and immunosuppressive effects, are the primary infiltrating immune cells (Filipazzi et al., 2012; Marvel and Gabrilovich, 2015). It is anticipated that directing attention towards monocytes or other stromal components will alter the gliomas “cold” TME to a more “hot” TME phenotype. The efficiency of conventional first-line immunotherapy for glioma may be enhanced by this alteration (Tomaszewski et al., 2019). It may be possible to develop more effective therapeutic strategies if we understand the interaction between LGG and immune cells. Our findings using the TIDE algorithm supported the conclusion that C2 exhibited greater responsiveness to immunotherapy compared to C1. Furthermore, the SubMap method was employed to juxtapose the expression patterns of the LGG group with other previously published immune checkpoint datasets, which additionally validates the heightened susceptibility of C2 to immunotherapy, particularly PD-1.

The EMT-related survival prognosis model constructed by WGCNA and LASSO algorithm contains a series of hub genes, such as CLIC1, CTSA and SLC39A1. Multiple studies underscored the tumor-driver roles of the hub genes identified in present study. These hub genes were potential to be novel therapeutic targets and prognostic predictors in LGG. CLIC1, extensively studied within the CLIC family concerning tumors, has potential as a diagnostic indicator and therapeutic target. It has been linked to various cancers, influencing cell processes, including cell viability and mitochondrial function (Dehghan-Nayeri et al., 2017; Singha et al., 2018). In breast cancer, elevated CLIC1 expression correlates with tumor characteristics like size, TNM classification, grade, lymph node metastasis, and Ki67, while lower expression associates with extended OS and progression-free survival, suggesting its diagnostic potential (Xia et al., 2022). In esophageal squamous cell carcinoma (ESCC), CLIC1 is linked to clinical TNM classifications (Geng et al., 2022). CLIC1 knockdown in ESCC cell lines inhibits mTOR signaling, affecting cell proliferation and protein expression (Geng et al., 2022). High CLIC1 expression in lung adenocarcinoma predicts shorter overall survival and functions as an independent prognostic factor (Yasuda et al., 2022). In gastric cancer, CLIC1 absence impedes invasion and migration by affecting AMOT-p130 expression, possibly contributing to metastasis (Qiu et al., 2020). In hepatocellular carcinoma (HCC), upregulated CLIC1 is associated with aggressiveness, metastasis, and poor prognosis (Peng et al., 2020). GBM exhibits high CLIC1 expression (Setti et al., 2013). Reducing CLIC1 expression impairs cell proliferation and self-renewal in GBM, while CLIC1-mediated channel activity correlates with tumor aggressiveness (Setti et al., 2013). CLIC1 modulates reactive oxygen species and pH in human GBM stem cells, impacting motility and proliferation, making it a potential therapeutic target (Peretti et al., 2018). The study by Biasiotta et al. identified CLIC1 among nine genes with significant alterations in ion channels in solid tumors and vascular malformations, particularly in GBM and bladder cancers (Biasiotta et al., 2016). CLIC1 expression is correlated with the drug-resistant protein MRP1. Knockdown of CLIC1 in human choriocarcinoma cell lines reduces MRP1 expression (Wu and Wang, 2016). Additionally, CLIC1 has been found to transfer from GBM cells to microvascular epithelial cells through extracellular vesicles, potentially impacting metastasis (Thuringer et al., 2018). In summary, CLIC1 plays a crucial role in various cancers, including breast cancer, ESCC, lung adenocarcinoma, gastric cancer, and HCC, making it a potential target for cancer treatment due to its influence on cell proliferation, migration, invasion, and metastasis. Research into CLIC1’s role in cancer and glioma progression and patient survival is promising, but further studies are needed to fully understand the mechanisms of action and develop targeted therapies.

Research on HCC has shown that SLC39A1 overexpression is linked to immune infiltration and promotes tumor progression (Zhang et al., 2021). Yuan et al. (2022) conducted a study to explore SLC39A1’s potential as a tumor suppressor in renal cell carcinoma (RCC) using integrated omics analyses. They found that SLC39A1 significantly impacts various metabolic pathways and triggers communication among multiple signaling pathways. This research provides valuable insights into RCC development and the molecular changes induced by SLC39A1 (Yuan et al., 2022). Furthermore, glioma tissues exhibit increased SLC39A1 expression, strongly associated with clinical features like grade, IDH mutation status, and 1p19q co-deletion status. Elevated SLC39A1 levels are linked to reduced survival chances and contribute to glioma malignancy by promoting cell growth, inhibiting cell death, and influencing immune cell infiltration in the tumor microenvironment (Wang et al., 2020). SLC39A1 shows promise as a novel prognostic biomarker and therapeutic target for gliomas.

CTSA, a lysosomal protease, is upregulated in various cancers, including HCC and prostate cancer (Park et al., 2020; Wang et al., 2021; Luo et al., 2022). Its role as a potential diagnostic and prognostic biomarker has been explored. Petrera et al. (2016) demonstrated that inhibiting CTSA in a mouse model improved cardiac functionality in heart failure (Petrera et al., 2016). High CTSA levels can differentiate HCC from healthy liver tissue and are linked to poorer survival rates (Wang et al., 2021). Luo et al. (2022) found high CTSA expression correlated with poor HCC patient outcomes, reinforcing its prognostic value (Luo et al., 2022). In prostate cancer, Park et al. (2020) showed that suppressing CTSA gene expression reduced proliferation, migration, and tumorigenesis (Park et al., 2020). CTSA is a potential therapeutic target and prognostic biomarker in various cancers. Hu et al., Zhang et al., Toss et al., and Kim et al. explored CTSA’s role in lung adenocarcinoma (Hu et al., 2020), glioma (Zhang et al., 2022), breast ductal carcinoma *in situ* (Toss et al., 2019), and canine inflammatory mammary adenocarcinoma (Kim et al., 2020), respectively. Hu et al. found that CTSA promotes lung adenocarcinoma progression and may be a promising target for treatment (Hu et al., 2020). Zhang et al. linked CTSA to poor prognosis in glioma (Zhang et al., 2022). Toss et al. associated CTSA with unfavorable outcomes in breast ductal carcinoma *in situ* (Toss et al., 2019). Kim et al. demonstrated the impact of leptin on inflammatory mammary adenocarcinoma in dogs by modulating CTSA expression (Kim et al., 2020). In summary, CTSA is significant in different cancers as a potential treatment target and predictor. Further research is needed to elucidate its precise molecular mechanisms and develop tailored treatments.

## 5 Conclusion

In conclusion, the present study comprehensively demonstrated the difference in molecular subtype of LGG based on EMT-related genes, thus revealing the tumor heterogeneity of LGG. We identified two distinct subtypes of LGG-EMT with varying clinical prognoses, clinicopathological characteristics, mutation statuses, immune cell infiltration, tumor microenvironment, and signaling pathway activities. The heterogeneity among LGG-EMT subtypes leads to divergent responses to immunotherapy and chemotherapy, guiding the precision treatment of LGG. Moreover, we developed and validated a reliable EMT-signature for LGG prognosis, and speculated about several small molecule compounds that could enhance the clinical practical value of LGG. Further experimental studies can help us understand the underlying mechanisms of EMT in LGG, thereby providing additional support for the clinical utility of the prognostic signature.

## Figures and Tables

**Table:** 

**CAMs**	cell adhesion molecules
**CDF**	cumulative distribution function
**CGGA**	the Chinese Glioma Genome Atlas
**CLIC1**	Chloride Intracellular Channel 1
**CNA**	copy number alterations
**CTA**	Cancer Testicular Antigens
**CTRP**	Cancer Therapeutics Response Portal
**DCIS**	ductal carcinoma *in situ*
**DEGs**	differentially expressed genes
**DSS**	disease-specific survival
**ECM**	extracellular matrix
**EMT**	epithelial-mesenchymal transition
**ESCC**	esophageal squamous cell carcinoma
**ESTIMATE**	Estimation of Stromal and Immune cells in MAlignant Tumours using Expression
**FDR**	false discovery rate
**GBM**	glioblastoma
**GDAC**	Genome Data Analysis Center
**GDSC**	Genomics of Drug Sensitivity in Cancer
**GEPIA**	gene expression profiling interactive analysis
**GS**	gene significance
**GSVA**	gene set variation analysis
**HCC**	hepatocellular carcinoma
**IC50**	half-maximal inhibitory concentration
**ICB**	immune checkpoint blockade
**KM**	Kaplan-Meier
**LGG**	low-grade glioma
**MM**	module membership
**MSigDB**	Molecular signatures database
**OS**	overall survival
**PCA**	principal components analysis
**RCC**	renal cell carcinoma
**ROC**	receiver operating characteristic
**RT-qPCR**	Real-time quantitative PCR
**scRNA-seq**	single cell RNA-sequencing
**SNP**	single-nucleotide polymorphisms
**ssGSEA**	single-sample gene set enrichment analysis
**TAD**	topological associating domain
**TCGA**	the Cancer Genome Atlas
**TIDE**	tumor immune dysfunction and exclusion
**TIICs**	tumor infiltrating immune cells
**TME**	tumor microenvironment
**TMZ**	temozolomide
**TNM**	tumor node metastasis
**TPM**	transcripts per kilobase million
**t-SNE**	T-distributed stochastic neighbor embedding
**WGCNA**	weighted correlation network analysis

## Data Availability

The original contributions presented in the study are included in the article/Supplementary Material, further inquiries can be directed to the corresponding author.
