# Supplementary figures and images for: Integrating bulk and single-cell RNA sequencing data reveals epithelial-mesenchymal transition molecular subtype and signature to predict prognosis, immunotherapy efficacy, and drug candidates in low-grade gliomas
Source: Front Pharmacol. 2023 Nov 20;14:1276466. doi: 10.3389/fphar.2023.1276466 (PMC10694300; doi:10.3389/fphar.2023.1276466)

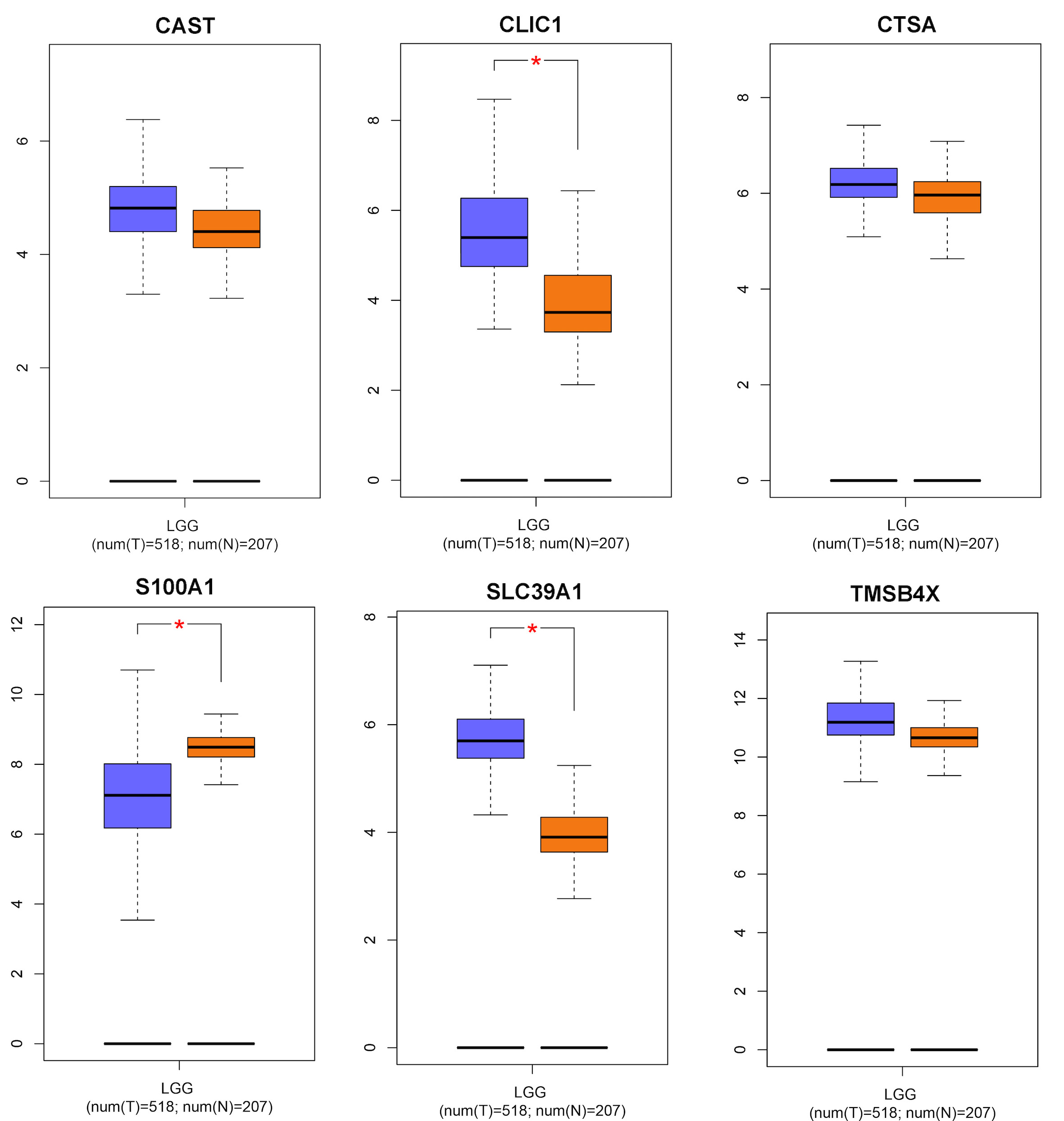

Supplement: Supplementary file 1 [file Image3.JPEG]

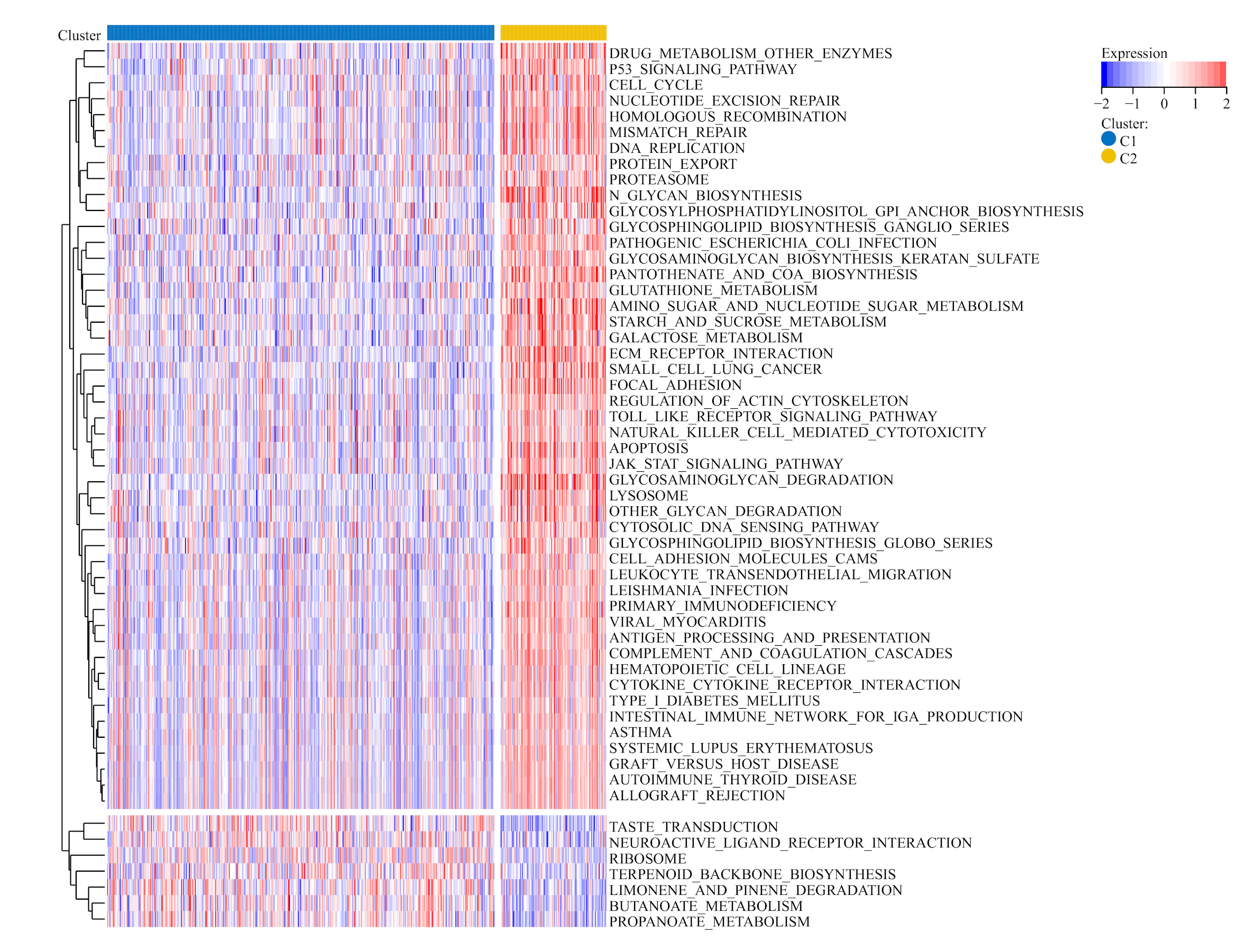

Supplement: Supplementary file 2 [file Image1.JPEG]

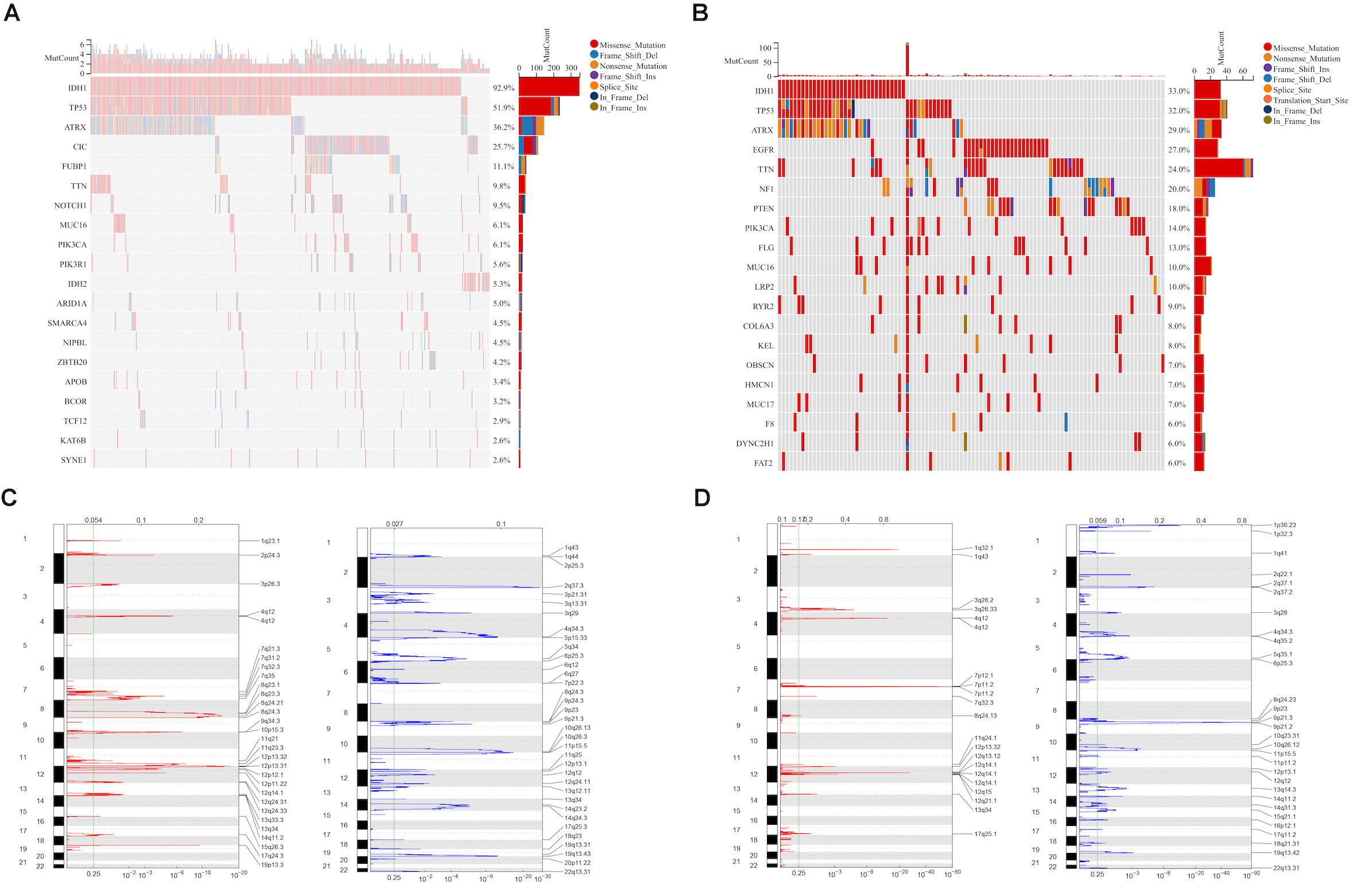

Supplement: Supplementary file 3 [file Image2.JPEG]
